# The effects of alcohol dependence on the CSF proteome in mice: Evidence for blood-brain barrier dysfunction and neuroinflammation

**DOI:** 10.1016/j.nbd.2025.107254

**Published:** 2025-12-30

**Authors:** Natalie P. Turner, Michal Bajo, Amanda J. Roberts, Marisa Roberto, John R. Yates

**Affiliations:** a The Scripps Research Institute, 10550 North Torrey Pines Rd, La Jolla, CA 92037, United States of America; b Animal Models Core Facility, The Scripps Research Institute, 10550 North Torrey Pines Road, La Jolla, CA 92037, United States of America

**Keywords:** Alcohol, Cerebrospinal fluid, Proteomics, Neuroinflammation, Blood-brain barrier

## Abstract

Alcohol use disorder (AUD) represents a significant neurological health burden, yet the biological mechanisms underlying alcohol-induced brain pathology remain incompletely understood. Moreover, the molecular underpinnings of the transition from alcohol exposure to alcohol dependence are not well-characterized. We used mass spectrometry (MS)-based proteomics in a preliminary discovery study to compare cerebrospinal fluid (CSF) of alcohol-exposed Non-dependent (Non-dep) versus alcohol-dependent (Dep) mice that underwent the chronic intermittent ethanol (alcohol) – two-bottle choice (CIE-2 BCE) procedure and systemic anti-IL-6 Receptor antibody administration (*n* = 9; female = 5, male = 4). CSF samples from individual mice were processed for proteomic analysis and digested with trypsin overnight. Peptides were analyzed via data-independent acquisition (DIA)-MS and data were processed in DIA-NN at 1 % FDR. We identified 595 unique proteins across both groups, with 140 proteins differentially detected in CSF from Dep mice and 62 proteins specific to alcohol-exposed but Non-dep controls. The Dep-specific proteins revealed signatures of blood-brain barrier (BBB) disruption, neuroinflammation, cellular stress responses, and complement system activation. In contrast, Non-dep-specific proteins indicated preserved protective mechanisms including complement regulation, anti-inflammatory signaling, and neuronal calcium homeostasis. Ethanol-dependent-specific findings include MMP2, BIP, and to a lesser extent VE-cadherin (CDH5) and VCAM1, indicative of the beginnings of endothelial damage and BBB disruption, alongside established neuroinflammation markers GFAP, CHI3L1, and CX3CL1. This work provides novel preliminary protein-level evidence that alcohol exposure and alcohol dependence are dichotomous; despite the small sample size and limited power for moderate effect sizes, there appears to be a clear molecular transition from maintained protective mechanisms to vascular damage, BBB breakdown, and sustained neuroinflammation.

## 1. Introduction

Alcohol use disorder (AUD) leads to significant neurological impairment, including cognitive decline, increased neuroinflammation, and blood-brain barrier (BBB) dysfunction. Chronic alcohol exposure disrupts tight junction proteins through multiple pathways, including matrix metalloproteinase activation, oxidative stress, and novel purinergic signaling mechanisms (Muneer et al., 2012; Pimentel et al., 2020). These processes create a ‘leaky’ barrier that allows peripheral inflammatory mediators to enter the brain, potentially establishing the neuroinflammatory cycles observed in alcohol dependence, which are comparable to that seen in other neurological conditions such as Alzheimer’s Disease (Lékó et al., 2023; Joshi et al., 2024).

There is accumulating evidence that cytokines can also be produced locally in the brain, offering an alternative hypothesis of compartmentalized neuroinflammation (Tyagi and Bartley, 2025). In the context of AUD, whether cytokine production in the CNS is similar between acute versus chronic ethanol (EtOH) exposure is not well-defined. For example, in transgenic mouse models of increased astrocyte-produced IL-6, neuroadaptations in the hippocampus were found to be mediated by IL-6 following acute alcohol exposure, implicating IL-6-mediated mechanisms in the development of alcohol-dependence (Hernandez et al., 2016; Roberts et al., 2019). In a related study, the same transgenic mouse line was used to examine the protein-level effects of IL-6 on its signal transduction partners Signal Transducer and Activator of Transcription (STAT3), CCAAT-enhancer binding protein (C/EBP) beta, and p42/p44 mitogen-activated protein kinase (MAPK) in cerebella of naïve and EtOH-dependent mice (Gruol et al., 2021). IL-6 and CIE EtOH exposure/withdrawal were both found to affect protein levels of IL-6 transduction partners, suggesting a similar mechanism between the two stimuli within the cerebellum as well as a more general role for IL-6 in disorders of the CNS (Gruol et al., 2021). In recent studies, using a chronic intermittent ethanol vapor – two-bottle choice (CIE-2 BCE) paradigm (Roberts et al., 2019; Lopez and Becker, 2014; Patel et al., 2021; Salem et al., 2024; Borgonetti et al., 2025; Borgonetti et al., 2023) we generated non-dependent mice (Non-dep) that drink moderate amounts of alcohol, and dependent mice (Dep), whose alcohol intake escalates following EtOH vapor exposure, and found sex-dependent increased IL-6 protein in the spinal cord of dependent mice that developed EtOH-induced mechanical allodynia (Borgonetti et al., 2025; Borgonetti et al., 2023). However, systemic treatment of an IL-6 receptor antibody (IL-6R Ab) did not reduce alcohol-associated pain sensitivity in the same mouse model of alcohol dependence used in the current studies (Borgonetti et al., 2025).

In addition to evidence from preclinical studies, recent neuroimaging and cerebrospinal fluid studies have confirmed that AUD can be characterized by activated microglia, elevated cytokines, and complement system dysregulation (Adams et al., 2023; Azuar et al., 2021). However, the molecular mechanisms distinguishing chronic alcohol exposure with moderate alcohol drinking from chronic alcohol exposure with excessive alcohol drinking that is characteristic of alcohol dependence remain poorly understood. Current biomarker approaches focus primarily on hepatic dysfunction and chronic consumption detection (Niemelä, 2016), leaving a significant gap in neurological diagnostic and prognostic tools. Studies on the distinct mechanisms that affect BBB integrity and promote increased barrier permeability or breakdown associated with alcohol-dependence, as opposed to acute EtOH exposure, are also currently limited (Michinaga and Koyama, 2019; Vore and Deak, 2021; Becker, 2008).

Proteomic analysis of cerebrospinal fluid (CSF) offers a unique window into brain pathology by allowing detection of proteins normally confined to specific brain regions. Advances in biomarker research have established several proteins as reliable indicators of neurological damage, including glial fibrillary acidic protein (GFAP) for astrocyte activation (Eng and Ghirnikar, 1994) and ubiquitin C-terminal hydrolase L1 (UCHL1; gene = *Park5*) for neuronal injury (Mi and Graham, 2023). Despite these advances, a fundamental question remains unanswered: does alcohol dependence represent a quantitative progression from exposure, or does it involve qualitatively distinct pathophysiological mechanisms? This distinction has critical implications for early intervention strategies and biomarker development.

To address this question, we systematically evaluated and compared CSF samples collected from Non-dep and Dep mice across six 2 BCE/CIE-2 BCE cycles by mass spectrometry (MS)-based proteomics. As all mice were administered IL-6R antibody injections in the final week (2 BCE) of EtOH exposure, we were able to determine whether alternative mechanisms of neuroinflammation independent from the IL-6 pathway are activated in alcohol dependence vs Non-dep alcohol exposure. The CSF proteome of 2 BCE Non-dep mice was distinct from Dep mice, which contained proteins associated with neuroinflammation, compromised immune surveillance and loss of protective function. Additionally, the identification of the clinically validated biomarker of astrocyte activation, GFAP, and the protein Matrix Metalloproteinase 2 (MMP2) associated with BBB breakdown, in Dep mice supports the hypothesis that BBB disruption and immune dysregulation is unique to alcohol dependence.

## 2. Results and discussion

### 2.1. Proteomic profiles differ between alcohol-dependent and non-dependent mice

**Fig. 1.**
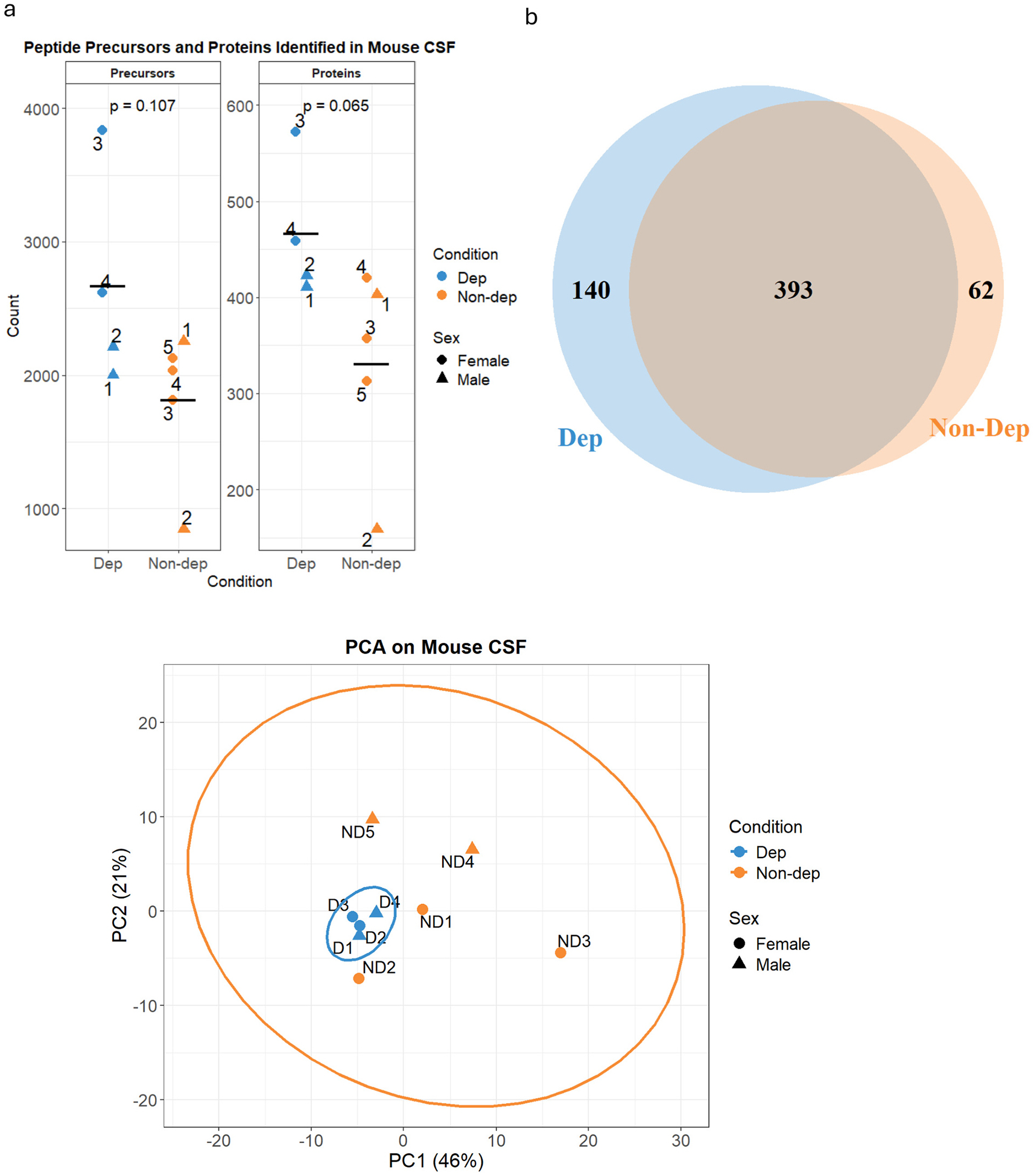
Proteomic analysis of Mouse CSF. a) Peptide precursors (left) and proteins (right) identified in Non-dep (orange; *n* = 5) and Dep groups (blue; *n* = 4) with individual replicate labeling. Horizontal lines represents the mean value. Displayed *p*-values were calculated using Student’s *t*-test. b) Venn diagram of proteins identified in Non-dep and Dep groups; Dep unique = 140; Non-dep unique = 62; overlap = 393. c) Principal component analysis (PCA) plot with 95 % confidence ellipse of Non-dep (orange) and Dep (blue) mice. Dep mice show clustering regardless of sex, whereas Non-dep mice are more dispersed, although segregate by sex along the PC2 axis. Circles = females; triangles = males.

Data-independent acquisition (DIA)-MS enables proteomic analysis of complex samples through simultaneous fragmentation of all peptides within a specific mass-to-charge ratio (*m*/*z*) isolation ‘window’, and sequential “stepping” through the entire MS1 mass range (e.g., 400–1200 *m*/*z*) with wide sequential windows (5–25 *m*/*z)* (Venable et al., 2004). Because DIA is used to fragment all peptides within each isolation window rather than preferentially selecting the top N most abundant peptide precursors for fragmentation (as in data-dependent acquisition; DDA), this approach was selected for the current study to mitigate the possibility of missing peptide features. If features are missing in a DIA experiment, it is likely due to extremely low peptide abundance; thus, DIA provides a more confident analysis of qualitative datasets than in DDA experiments, where peptide features can be missing at random. Proteomic analysis using DIA-MS identified an average of 1816 peptide precursors and 330 proteins in the alcohol-exposed control group (Non-dep) at 1 % FDR (Fig. 1a). In the ethanol dependent group, an average of 2667 peptide precursors and 466 proteins were identified. One Non-dep animal (ND2) yielded fewer precursor and protein identifications than other samples, likely reflecting suboptimal sample preparation rather than biological variation. Further analysis found 140 proteins preferentially detected in alcohol-dependent mice and 62 proteins preferentially detected in Non-dep mice (Fig. 1b). Given the limited statistical power for detecting moderate effect sizes (38 % power for 75 % vs 20 % detection rates in Dep vs Non-dep, respectively), the 140 Dep-specific and 62 Non-dep-specific proteins likely represent minimum estimates of the true proteomic differences between groups. A principal component analysis (PCA) plot with 95 % confidence ellipse was generated to assess the relationships between replicates within and between groups (Fig. 1c). PCA preserves the overall data structure while performing dimensionality reduction, enabling visualization of the relationships between data points (replicates) in two dimensions. The Dep animals formed a distinct cluster regardless of sex (orange points), whereas the Non-dependent animals were dispersed across the plot but could be distinguished along the PC2 axis by sex. This pattern suggests that the CSF proteome more strongly reflects the dependence state than baseline sexual dimorphism or individual variation in drinking behavior, supporting its utility as a biomarker of alcohol dependence. The protein profiles of both groups were interrogated further using enrichment analysis and STRING network analysis to identify putative molecular mechanisms associated with alcohol-dependence.

**Fig. 2.**
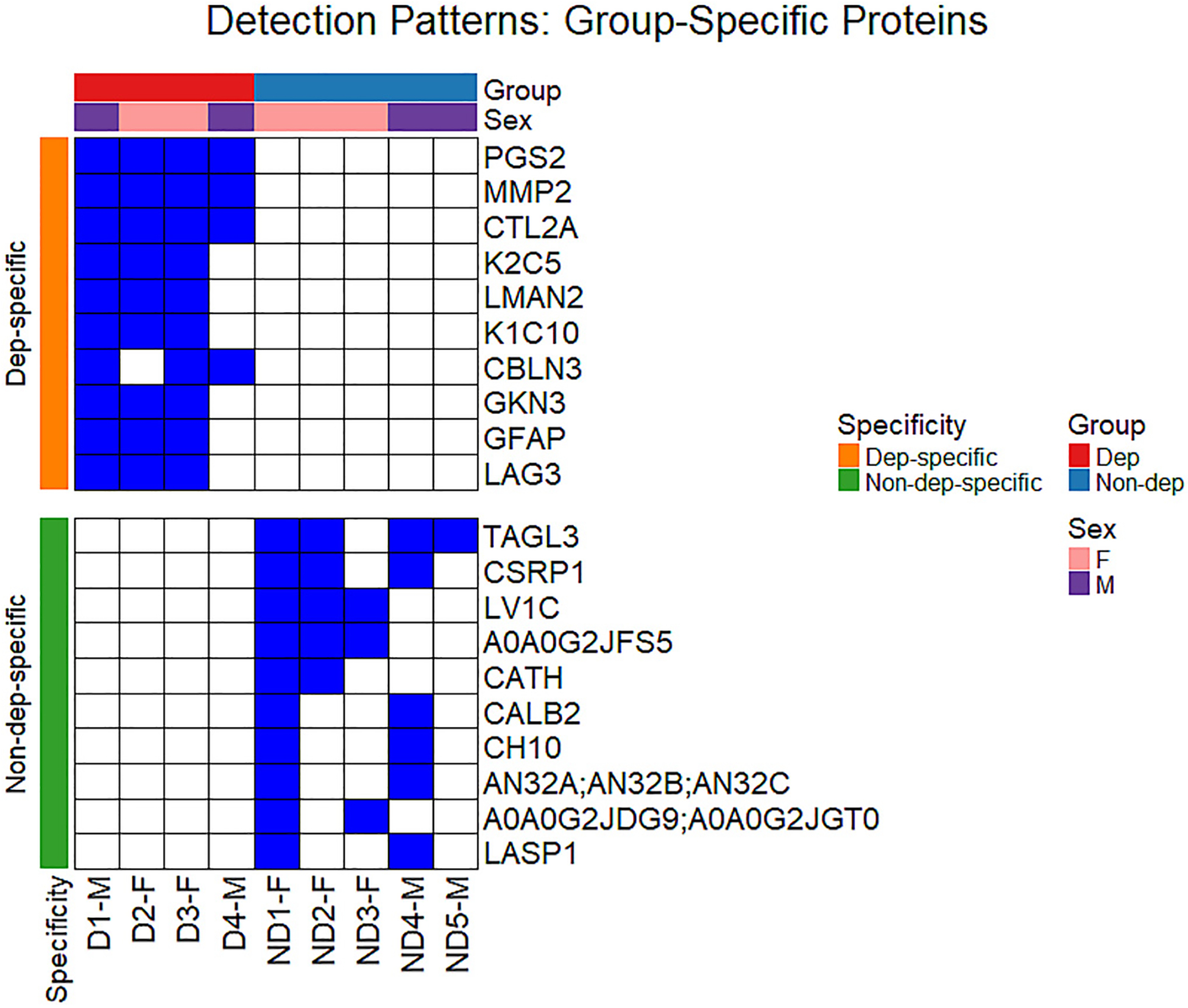
Detection Patterns of Group-Specific Proteins in Mouse CSF Samples. Heatmap showing the top 10 group-specific proteins detected (blue) or not-detected (white) across experimental samples. Samples were analyzed by data-independent acquisition (DIA) MS, therefore missing values are likely to represent protein levels below the lower limit of detection (i.e., not missing at random). Group specificity for the top 10 proteins was determined by protein detection in ≥2 of the replicates within each group and 0 replicates of the opposite group.

The top 10 group-specific proteins are shown in Fig. 2. The heatmap displays segregation of groups based on treatment-type, demonstrating how a shift to alcohol dependency is reflected in the CSF proteome. Numerous proteins detected preferentially in the Dep group are associated with neuroinflammation (GFAP), and with synaptic and extracellular matrix (ECM) remodeling (Reelin – RELN; MMP2). The preferential detection of proteins such as CALB1 (Calbindin-1), SUMO2/SUMO3, and TAGL3 (Transgelin-3) suggests functional homeostatic and neuroprotective mechanisms are maintained in the Non-dep group. Additionally, detection patterns of proteins in immunoglobulin variable regions (KV5AB, KV5A7, LV1C, HVM02/03) indicate that immune surveillance may be lost in the Dep group. (For the full list of filtered protein identifications, see Supplementary file 1.)

### 2.2. Classification of proteins associated with alcohol dependence

The following criteria were used to classify protein-level evidence of alcohol dependence or non-dependence, based on detection patterns in mouse CSF samples:

#### Strong Evidence:

- Dep-preferred: ≥3/4 (≥75 %) Dep AND ≤ 1/5 (≤20 %) Non-dep
- Non-dep-preferred: ≥4/5 (≥80 %) Non-Dep AND ≤ 1/4 (≤25 %) Dep

#### Moderate – Weak Evidence:

- Dep-preferred: ≥2/4 (≥50 %) Dep, ≤1 Non-dep
- Non-dep-preferred: ≥3/5 (≥60 %) Non-dep, ≤1 Dep
- Weak: Detected in <50 % of preferred group or ≥ 2 in both groups

Dep-preferred and Non-dep-preferred proteins with strong-moderate evidence are listed in Tables 1 and 2.

The proteomic signatures identified here represent minimum estimates of alcohol dependence-associated changes, as our study design provided adequate power only for detecting large effect sizes. This suggests that the true extent of proteomic alterations in alcohol dependence may be substantially greater than reported.

### 2.3. Non-dep-specific CSF proteins indicate preservation of protective mechanisms

Proteins unique to the Non-dep ethanol group revealed several protective and regulatory mechanisms that appear to be lost in alcohol dependence. CALB1, a critical calcium-binding protein primarily expressed in inhibitory neurons, was preferentially detected in Non-dep (1/4 Dep vs 4/5 Non-dep). In a *Calb1*^−*/*−^ KO mouse model, *Calb1* was found to regulate sex-specific behavioral differences. KO was related to reduced anxiety in both sexes; however, fear, memory and social behavior changes were observed in male but not female mice (Harris et al., 2016). The same study also found an effect on histone deacetylase transcript levels (*Hdac3* and *Hdac4*) in the amygdala and prefrontal cortex (PFC) between WT and KO animals, suggesting a role for CALB1 in epigenetic regulation within these brain regions (Harris et al., 2016). CALB1 has also garnered interest for its neuroprotective properties in neurodegenerative conditions such as Alzheimer’s (Odero et al., 2010) and Parkinson’s Disease (Brimblecombe et al., 2019). Preserved calcium homeostasis proteins (CALB1; Calretinin-2, CALB2) in Non-dep mice suggest that neuronal calcium buffering capacity is maintained, which may protect against excitotoxicity and cell death (Gattoni and Bernocchi, 2019; Schwaller, 2014; Fairless et al., 2019). The loss of these protective mechanisms in dependence could predispose neurons to calcium-mediated damage.

The preferential detection of numerous proteins associated with cellular stress response and protein degradation in Non-dep animals suggests that protein modification systems crucial for cellular stress responses are maintained. SUMO2/SUMO3 conjugation proteins (1/4 Dep vs 3/5 Non-dep) are associated with neuroprotective mechanisms (Datwyler et al., 2011), and it has been suggested that they act through multiple mechanisms to carry out protein quality control in the brain. The downregulation of Transgelin-3 (TAGL3; 0/4 Dep vs 4/5 Non-dep), a regulator of cytoskeletal regulation and smooth muscle function, has been implicated as a driver of neuroinflammation in astrocytes (Arnaud et al., 2022). Multiple specific immunoglobulin variable regions were also preferentially detected in the Non-dep group, suggesting that adaptive immune surveillance mechanisms may be compromised in alcohol dependence. These included kappa light chain variable regions (KV5AB cluster: 1/4 Dep vs 4/5 Non-dep; KV5A7: 0/4 Dep vs 2/5 Non-dep), lambda light chain variable regions (LV1C: 0/4 Dep vs 3/5 Non-dep), and heavy chain variable regions (HVM02/03 and HVM57: 0/4 Dep vs 2/5 Non-dep each). The detection of these antibody components in the Non-dep group may be associated with immunosuppression and reduced adaptive immune capacity in alcohol dependence (Pasala et al., 2015).

### 2.4. Blood-brain barrier disruption in alcohol dependence

The BBB is a layer of tightly packed semi-permeable cells that act as gatekeepers to the CNS; the BBB selectively allows or disallows entry of molecules from systemic circulation into the neural compartment. Chronic alcohol exposure can disrupt the BBB, leading to increased neuroinflammation and cognitive decline (Wei et al., 2021; Togre et al., 2024). A significant finding in this study was the detection of MMP2 in all Dep mice but absence in the Non-dep group. MMP2 is an enzyme critically involved in ECM degradation and BBB breakdown, making its exclusive presence in dependent mice suggestive of vascular compromise (Rempe et al., 2016). The endoplasmic reticulum chaperone BiP (BIP; gene = *Grp78*/*Hspa5*) also displayed a strong Dep preference, with detection in all animals in the Dep group and 1/5 Non-dep mice. BIP was found to be associated with both endoplasmic reticulum dysfunction and the disruption of endothelial tight junctions in vitro, as its abundance was upregulated following ethanol exposure in a rat brain endothelial cell line, RBE4, and coincided with increased oxidative stress (Carrino et al., 2021). In humans, elevated levels of MMP2 can be detected in blood serum of individuals with alcoholic liver disease and was found to correlate with the development of liver cirrhosis (Prystupa et al., 2015). Similarly, increased BIP levels were found to be positively correlated with alcohol consumption grade and reflected the degree of alcohol-induced damage in human liver biopsy samples (Gaither et al., 2025). A less pronounced but consistent pattern could be observed with the tendency toward Dep-detection for endothelial junction proteins including VE-cadherin (CADH5; 2/4 Dep vs 1/5 Non-dep) and vascular cell adhesion molecule-1 (VCAM1; 2/4 Dep vs 0/5 Non-dep), which may indicate early signs of cellular response to vascular endothelial damage and compromised BBB function (Zhang et al., 2024; Gavard and Gutkind, 2008). As MMP2 and BIP are also considered markers of liver damage, it’s possible that their presence in CSF may originate in part from the liver, but due to increased BBB permeability these proteins have crossed into the neural compartment and thus can be detected in CSF. However, our CIE-2 BCE paradigm has not been typically associated with liver damage, and other alcohol models are used to induce alcohol-related liver injury (Cao et al., 2025). In either case, the strong detection of MMP2 and BIP in CSF from Dep mice suggests that the BBB is compromised in alcohol dependence.

Evidence for pericyte involvement was demonstrated by the Dep-preferred detection of alpha-smooth muscle actin (ACTA) in 3/4 dependent mice vs only 1/5 Non-dep mice. ACTA is a major contractile protein normally confined within pericytes (Brown et al., 2019), and its presence in CSF suggests pericyte detachment or death, a hallmark feature of BBB dysfunction. This finding suggests that alcohol dependence involves the pericyte component of the neurovascular system responsible for BBB regulation.

The ethanol dependent-preferred detection of laminin beta-2 (LAMB2) in 3/4 dependent animals versus only 1/5 Non-dep is suggestive of compromised basement membrane integrity in alcohol-dependent mice and suggests structural damage to the neurovascular unit (Zapata-Acevedo et al., 2022). The detection of fibulin-4 (FBLN4) in all dependent mice but only 1/5 Non-dep, and fibulin-5 (FBLN5) in 3/4 Dep mice but no Non-dep, could indicate leakage of these vascular scaffolding proteins from the ECM. Since FBLN4 and FBLN5 are essential for maintaining blood vessel strength and elasticity (Horiguchi et al., 2009; Spencer et al., 2005), their presence in CSF suggests vascular matrix degradation and compromised vessel integrity in alcohol dependence.

Within the dependent group, the log2 intensities of Dep-specific proteins showed positive associations with behavioral indices of dependence: LAMB2 correlated with CIE drinking, while GFAP correlated with drinking escalation ratio (Supplementary file 4, Fig. S3). Interestingly, MMP2 showed a sex-specific pattern, correlating with CIE drinking in males but not females. While limited sample size precludes formal statistical testing, these observations highlight potential sex differences in dependence-associated mechanisms. However, the binary detection of these proteins in Dep as opposed to Non-dep animals underlines their value as dependence-associated markers, regardless of quantitative correlations.

### 2.5. Neuroinflammation

The presence of several key markers in the alcohol dependent group is indicative of dependence-associated neuroinflammation. GFAP, the gold-standard biomarker for astrocyte activation, was detected in 3/4 Dep animals but 0/5 Non-dep, representing one of the strongest group differences observed. GFAP has been extensively validated as a biomarker for traumatic brain injury and neuroinflammation (Abdelhak et al., 2022), and cortical astrocyte activation has been linked to the regulation of ethanol consumption in mice (Erickson et al., 2021). These findings highlight the potential of GFAP as an indicator of chronic excessive alcohol exposure that induces dependence or related neurological damage.

Similarly, Chitinase-3-like protein 1 (CHI3L1) showed ethanol dependence preference (3/4 Dep vs 1/5 Non-dep). CHI3L1 is primarily secreted by activated astrocytes and has been established as a reliable biomarker for inflammatory CNS conditions. CHI3L1 levels in CSF have been associated with neuroinflammation across multiple neurological diseases, including multiple sclerosis and Alzheimer’s disease (Lananna et al., 2020). The preferential detection of CHI3L1 in dependent mice is suggestive of neuroinflammation; however, further studies are required to validate its association with the transition to alcohol dependence.

The chemokine CX3CL1 (fractalkine), which mediates microglial activation and neuroinflammation (Cardona et al., 2006), was also Dep-preferred (2/4 Dep vs 0/5 Non-dep). We detected other neuroinflammation-related proteins, including the immune checkpoint protein and regulator of cytokine production, lymphocyte activation gene 3 (LAG3), which was detected in 3/4 Dep animals and not detected in Non-dep. One study noted that an increase in soluble and membranous forms of LAG3 were observed in primary cultured microglia in vitro in response to interferon-γ (IFN-γ) treatment, with additional in vivo experiments confirming that LAG3 levels increase occurs via the IFN-γ/STAT1 pathway (Morisaki et al., 2023). LAG3 detection in dependent animals is consistent with the neuroinflammatory profile in the Dep group and supports the hypothesis that immune system dysregulation is driven by chronic alcohol consumption.

### 2.6. Alcohol-induced changes to the CSF proteome

The ethanol-dependence-specific proteins indicate a molecular response involving vascular damage, neuroinflammation, and cellular stress. The presence of VE-cadherin in CSF is particularly noteworthy, as this protein is normally tightly localized to endothelial junctions (Li et al., 2018), therefore its detection in the Dep group suggests BBB compromise. This finding aligns with recent mechanistic studies showing that alcohol disrupts VE-cadherin expression and BBB integrity through multiple pathways (Wei et al., 2021).

GFAP, CHI3L1, and CX3CL1 detection in dependent animals is consistent with a neuroinflammation signature suggestive of glial activation and CNS distress. The ability to detect these proteins within 4–5 weeks of dependence initiation offers critical insight into the rapid neurological decline that occurs following sustained alcohol exposure.

Immune System Dysregulation and Loss of Protective Mechanisms.

**Fig. 3.**
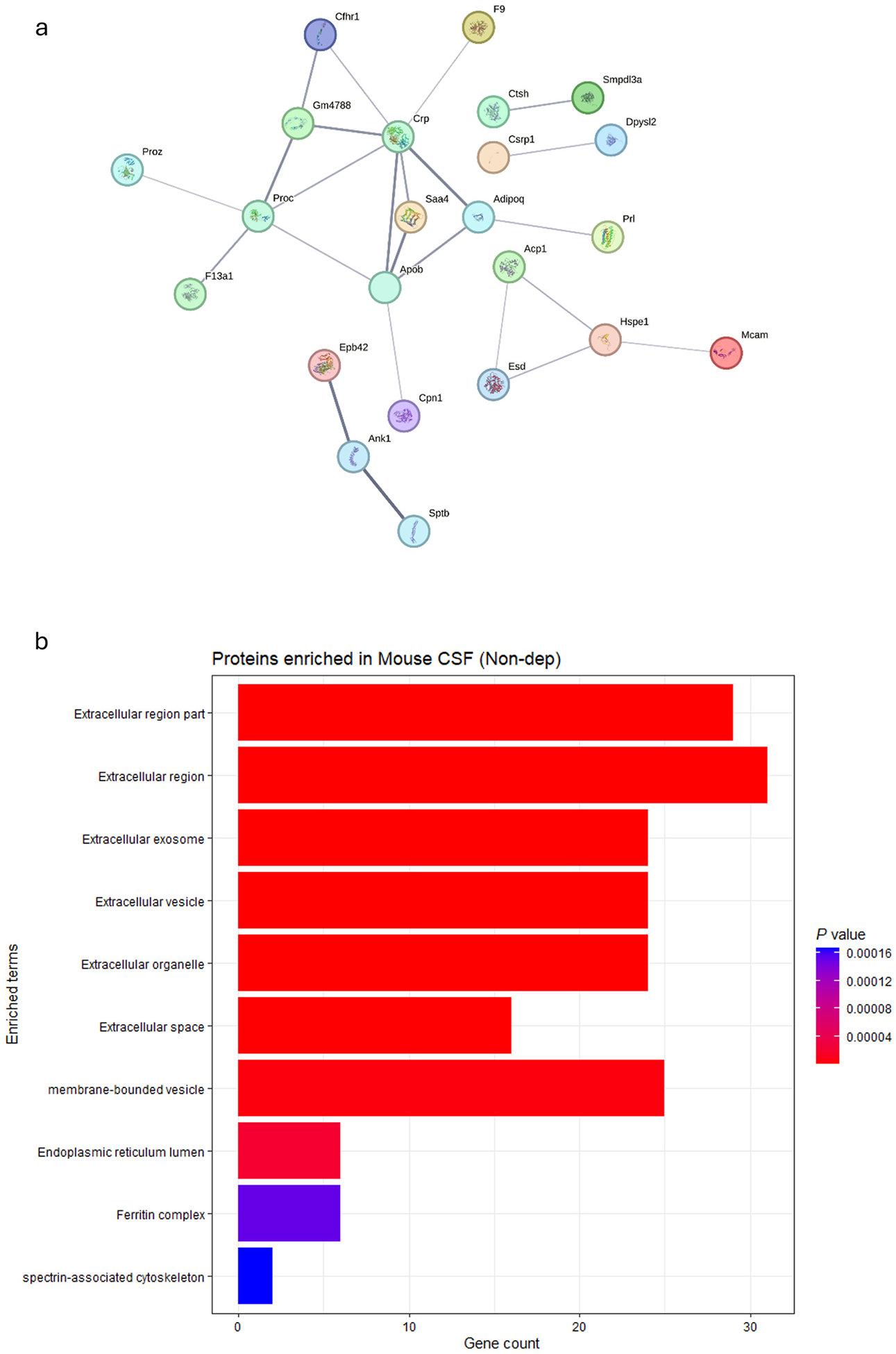
a) STRING protein-protein interaction (PPI) network analysis of proteins uniquely identified in alcohol-exposed, Non-dependent mice (Non-dep). Colored nodes: query proteins and first shell of interactors. Protein interactions were filtered at high confidence (≥0.700; line width is positively correlated with confidence level) and unconnected nodes removed. b) EnrichR analysis against Jensen COMPARTMENTS database shows enrichment of extracellular compartments among Non-dep-preferred proteins. Bar length represents gene count; color intensity indicates statistical significance (*P*-values).

STRING network analysis of Non-dep-preferred proteins revealed maintained physiological homeostasis mechanisms that appear to be down-regulated in alcohol dependence (Fig. 3a). The detection of proteins involved in acute phase response (CRP), and lipid transport (APOB) suggests preserved regulatory capacity in moderate alcohol-exposed Non-dep mice.

Analysis of Non-dep-preferred proteins against the Jensen COMPARTMENTS database revealed a markedly different pattern than Dep-specific proteins, suggesting maintained cellular organization and protective mechanisms in alcohol-exposed but Non-dep mice (Fig. 3b). While extracellular region proteins were enriched (~31 proteins), the overall extracellular protein diversity was substantially lower than observed in dependent mice (~95 proteins in the Dep group), suggesting dependent mice have more uncontrolled protein release. Importantly, Non-dep-specific proteins showed significant enrichment for protective complexes, including the ferritin complex, reflecting maintained iron homeostasis. The presence of endoplasmic reticulum lumen proteins suggests preserved cellular organization and protein processing capacity. This compartment profile supports the interpretation that alcohol-exposed Non-dep mice maintain protective mechanisms and cellular integrity, as opposed to the extensive extracellular protein leakage observed in dependent mice. The controlled extracellular protein presence in the Non-dep group likely represents normal physiological protein turnover rather than pathological cellular compromise.

**Fig. 4.**
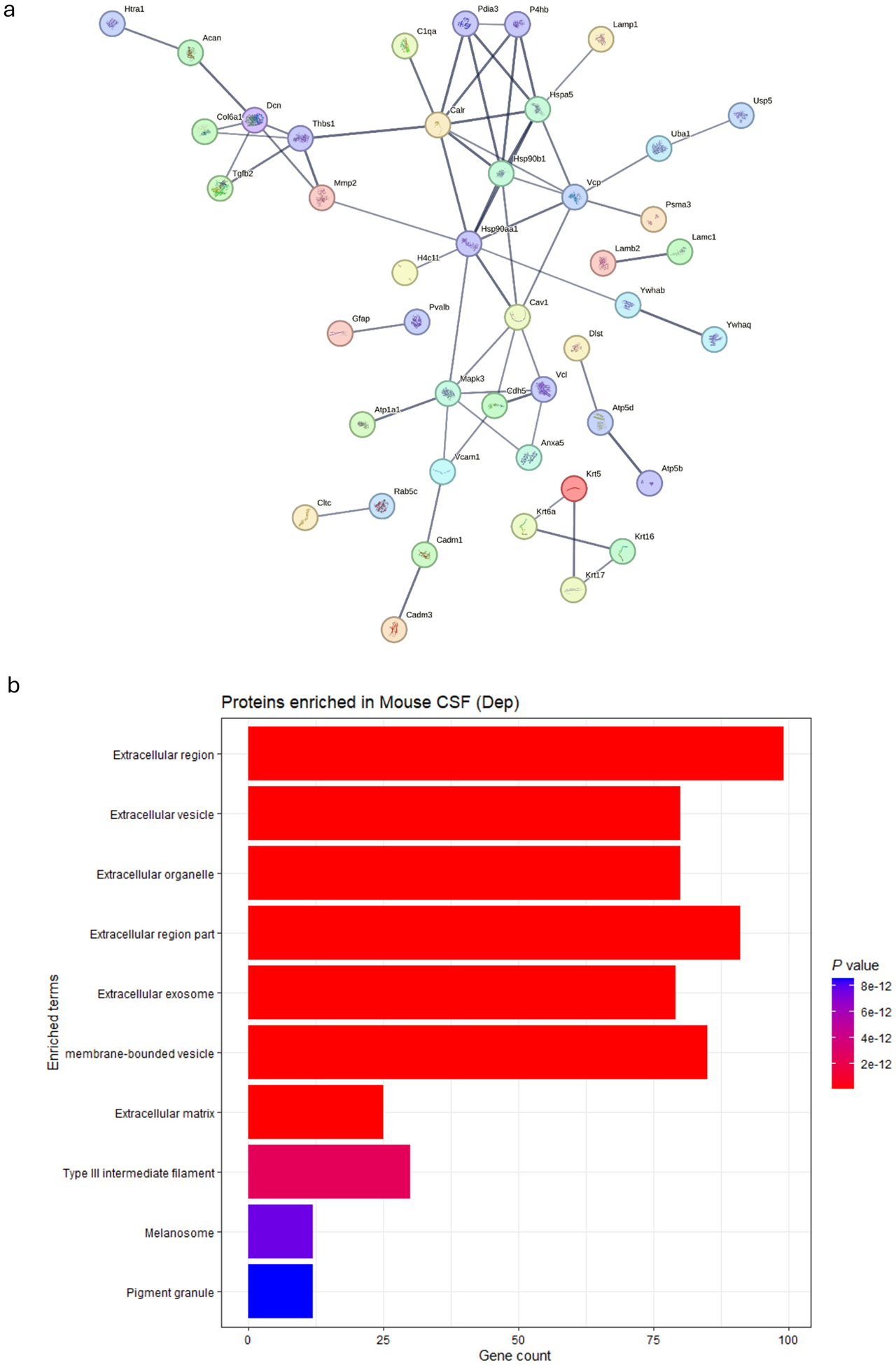
a) STRING protein-protein interaction (PPI) network analysis of proteins identified in alcohol-dependent mice (Dep). Colored nodes represent query proteins and their first-shell interactors. Protein interactions were filtered for high confidence (≥0.700; line width correlates with confidence level), and unconnected nodes were removed. PPIs between complement family member C1QA, heat shock proteins HSPA5 and HSP90B1, as well as proteasome and protein degradation proteins PSMA3, UBA1, and USP5, were scored as high to extremely high confidence (≥0.700–0.900). b) EnrichR analysis against the Jensen COMPARTMENTS database reveals significant enrichment of extracellular compartment proteins among Dep-preferred proteins. Bar length indicates gene count; color intensity reflects statistical significance (*P*-values).

In contrast, alcohol-dependent mice showed enhanced classical pathway activation, evidenced by the presence of C1QA and C6 proteins specifically in the Dep group (Fig. 4a). This represents dysregulated innate immune activation, while suppressed adaptive immunity is also evidenced in the same animals. C1QA activation of the classical complement pathway and C6 participation in membrane attack complex formation indicate both initiation and terminal complement activation, which are known contributors to neuroinflammation and synaptic pruning. Complement regulatory proteins (CFHR1, CFHR4) detected in the Non-dep group suggest that complement control is maintained (Skerka et al., 2013), which is juxtaposed with the complement activation (C1QA, C6) seen in dependent mice. This shift from regulated to dysregulated complement activation may contribute to neuroinflammation and tissue damage observed in models of alcohol dependence (Anand et al., 2023; Lowe et al., 2020; King et al., 2020). Dep-specific proteins also revealed a signature consistent with active barrier degradation and cellular injury. The Dep-enriched proteome contained BBB structural components being shed, including MMP2 (4/4 Dep, 0/5 Non-dep), the basement membrane protein LAMB2 (3/4 Dep, 1/5 Non-dep), endothelial junction protein VE-cadherin/CADH5 (2/4 Dep, 1/5 Non-dep), and the inflammation-associated adhesion molecule VCAM1 (2/4 Dep, 0/5 Non-dep). Collectively, the complement activation and BBB structural degradation signatures suggest that chronic alcohol dependence induces active barrier breakdown and neuroinflammation rather than simple increases in barrier permeability.

### 2.7. Cellular stress and protein quality control

Multiple indicators of cellular stress were found only in Dep mice. Heat shock proteins (HSPA5, HSP90B1) and protein disulfide isomerases (P4HB, PDIA3) suggest endoplasmic reticulum stress responses (Fig. 4a). Proteasome subunit alpha-3 (PSMA3) and ubiquitin-related enzymes (UCHL1, UBA1, USP5) show activation of the protein degradation system. These proteins indicate upregulation of the cellular stress response and attempts to handle cell and tissue damage but also links to misfolded protein accumulation and aggregation as proposed by others (Boukli et al., 2010; Gorini et al., 2014). Metabolic disturbance was evidenced by the presence of multiple ATP synthesis enzymes (ATP5B, ATP5D) and glycolytic enzymes (PFKAM; 2/4 Dep vs 0/5 Non-dep), suggesting mitochondrial dysfunction and energy metabolism disruption.

Overall, cellular compartment enrichment analysis provided evidence for structural compromise in the alcohol-dependent mice (Fig. 4b). The Dep-preferred proteins showed significant enrichment for extracellular compartments. This substantial extracellular protein presence, encompassing proteins associated with extracellular vesicles (EVs), organelles, and exosomes, suggests that alcohol dependence promotes increased leakage of cellular proteins into CSF while also promoting intercellular communication via EVs, which are known mediators of neuroinflammation in various neurological conditions (Cabrera-Pastor, 2024; Ruan et al., 2021). The enrichment of structural components, including Type III intermediate filaments, indicates that cellular integrity may be compromised, with cytoskeletal and membrane proteins detected in the extracellular space. These findings support the interpretation that in this model, alcohol dependence involves significant BBB dysfunction and cellular compromise, where normal compartmentalization between cellular and extracellular spaces becomes disrupted. The enhanced proteasome activity and cellular stress responses suggest that alcohol dependence overwhelms normal protein homeostasis mechanisms.

### 2.8. Functional pathway analysis

**Fig. 5.**
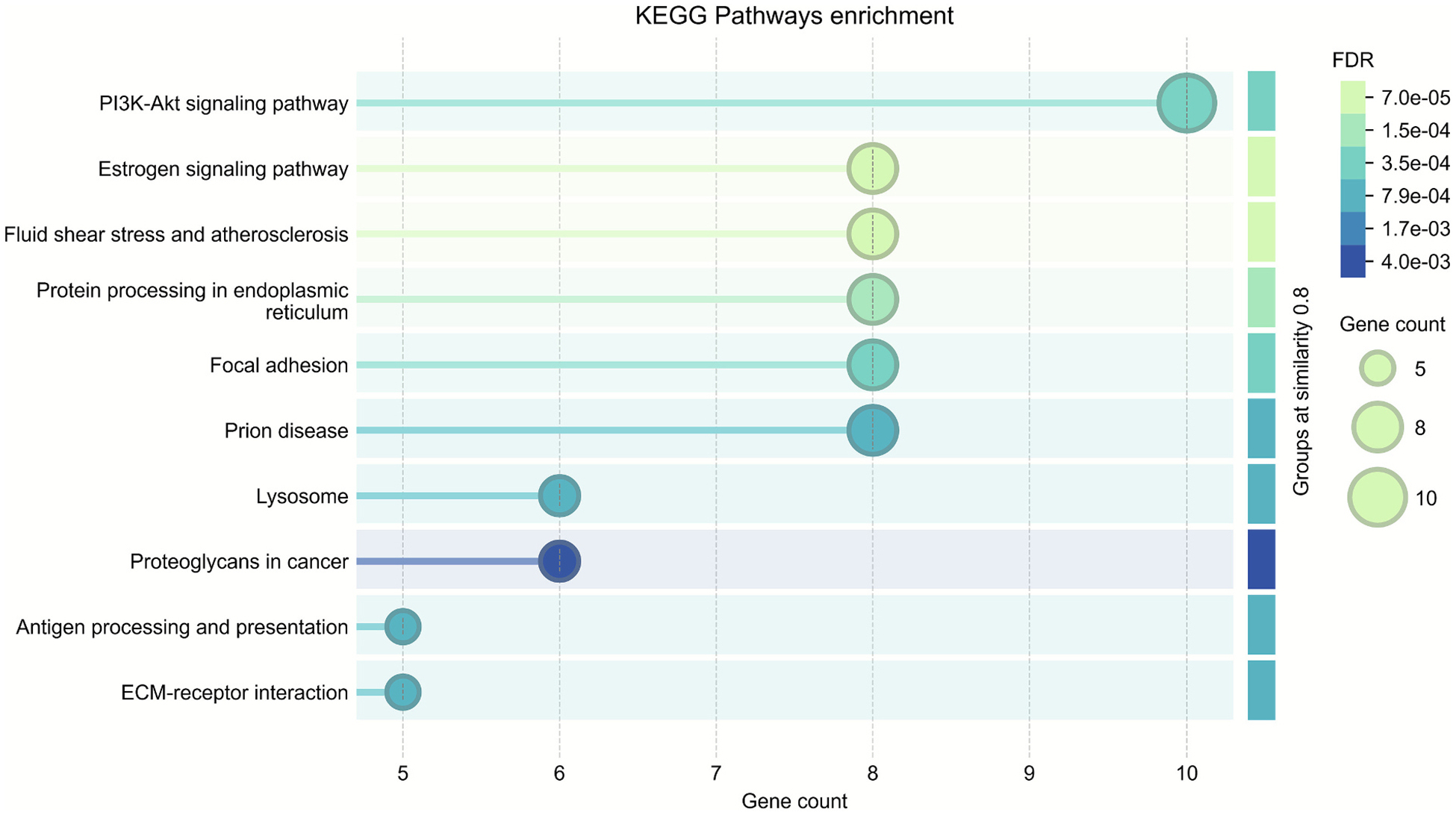
KEGG pathway enrichment analysis of Dep-specific proteins reveals multi-system dysfunction in alcohol dependence. Functional enrichment analysis was performed using STRING database on proteins detected in ≥2/4 alcohol-dependent and ≤ 1/5 alcohol-exposed Non-dep mice. Circle size represents gene count; color intensity indicates statistical significance (FDR values).

KEGG (Kyoto Encyclopedia of Genes and Genomes) pathway enrichment analysis of Dep-preferred proteins revealed significant enrichment of pathways consistent with the observed proteomic signatures (Fig. 5). The most significantly enriched pathway by gene count was PI3K-Akt signaling pathway (FDR = 3.5 × 10^−4^), indicating dysregulated cell survival and proliferation signaling that may contribute to altered neuronal responses to alcohol exposure. Prion disease pathway enrichment (FDR = 7.9 × 10^−4^) suggests protein misfolding and aggregation processes, consistent with the detected cellular stress response proteins. Fluid shear stress and atherosclerosis pathway involvement (FDR = 7.0 × 10^−5^) indicates vascular dysfunction patterns typically associated with cardiovascular disease, directly supporting the observed BBB breakdown evidenced by MMP2, VE-cadherin, and pericyte damage markers. Protein processing in endoplasmic reticulum pathway enrichment (FDR = 1.5 × 10^−4^) highlights the cellular stress response signatures, including the detection of heat shock proteins and protein quality control markers. Focal adhesion pathway involvement (FDR = 3.5 × 10^−4^) aligns with the detected extracellular matrix degradation and pericyte detachment, while estrogen signaling pathway enrichment (FDR = 7.0 × 10^−5^) may reflect loss of neuroprotective mechanisms. Collectively, these pathway enrichments suggest that alcohol dependence is characterized by dysregulated cellular signaling, protein homeostasis failure, vascular compromise, and loss of protective mechanisms.

### 2.9. Synaptic and neuronal dysfunction

Several proteins involved in synaptic function and neuronal integrity showed strong, but not exclusive, Dep preference. Neurofascin (NFASC), crucial for axon-glia interactions and node of Ranvier formation (Lananna et al., 2020), was detected in all Dep animals and 1/5 Non-dep (4/4 vs 1/5). RELN, critical for synaptic plasticity and neuronal migration (Beffert et al., 2005), showed identical preferential detection in dependent mice (4/4 vs 1/5).

Chondroitin sulfate proteoglycan 5 (CSPG5) was also Dep-preferred (4/4 vs 1/5). CSPG5 is involved in neural development and synaptic regulation (Pintér et al., 2020), and was found to be primarily expressed in GFAP+ radial glial cells in a transgenic mouse model focusing on neural stem cells and neural maturation (Tang et al., 2019). Neuronal pentraxin 1 (NPTX1) was detected in 3/4 Dep animals but absent in Non-dep. NPTX1 serves dual roles as both a proapoptotic protein induced by reduced neuronal activity and a regulator of Kv7.2 potassium channel surface expression, which modulates neuronal excitability (Figueiro-Silva et al., 2015). NPTX1 is also known for its role in regulation of AMPA receptor clustering and synaptic strength (Rennich et al., 2023). Taken together, these findings suggest that alcohol dependence disrupts synaptic organization and axon-glia communication.

## 3. Considerations and limitations

### 3.1. Contribution of alcohol pharmacokinetics to group differences

A consideration when interpreting these findings is whether proteomic differences between groups reflect dependence-specific neuroadaptations or pharmacokinetic differences from higher ethanol exposure in the CIE group. However, studies directly comparing equivalent ethanol doses in different temporal patterns demonstrate that neuroimmune consequences depend on dosing interval rather than total exposure (Gano et al., 2016), and key neuroadaptations occur only with intermittent exposure and withdrawal cycles, not continuous exposure at comparable concentrations (Reynolds et al., 2015). The “kindling” phenomenon, whereby repeated withdrawal episodes produce progressive neurobiological changes independent of cumulative dose (Becker, 1998), provides a mechanistic framework for the CIE-2 BCE model. Thus, while dose-dependent contributions cannot be entirely excluded, the proteomic signatures identified here are consistent with withdrawal-driven neuroadaptations characteristic of alcohol dependence (Becker, 2008; Uys et al., 2016; DePoy et al., 2013; Becker and Mulholland, 2014).

### 3.2. Sample quality

The presence of multiple keratin proteins (K2C5, K1C10, K2C1B) and hemoglobin (Hb) in the Dep-specific protein list raises questions about potential sample contamination or tissue damage. However, the biological pattern of neuroinflammation, BBB disruption, and synaptic dysfunction supports the biological significance of these findings rather than pure technical artefacts. We reviewed relevant literature that may shed light on the identification of these proteins by proteomics in mouse CSF and found one recent methodological study specifically aimed at CSF collection in murine models for the purpose of sample purity and contaminant mitigation (Lourbopoulos et al., 2025). Interestingly, even though that study (Lourbopoulos et al., 2025) reported undetectable levels of Hb in CSF samples with ELISA, proteomics analysis identified Hb, in addition to multiple classes of keratins, which were analyzed using an earlier version of the same DIA-NN pipeline (results available from ProteomeXchange, dataset identifier: PXD053568) (Lourbopoulos et al., 2025). Overall, the number of protein IDs were similar between our study and this comparator methodological study. This demonstrates that MS-based proteomics can detect trace amounts of these proteins below the detection limits of conventional immunoassays as well as supporting the legitimacy of these identifications outside of contamination. In the current study, the CSF proteome complexity was mostly similar across all samples (Fig. 1), which would likely not be the case if contamination had contributed significantly to the proteomic findings in the Dep group, although we do expect some variability due to inherent limitations associated with MS sample preparation (Klont et al., 2018; Varnavides et al., 2022). Given that numerous proteins indicate BBB leakage and vascular damage, further studies should focus on confirming these findings and any associated processes which could lead to cell and tissue breakdown or leakage into CSF. Increasing the sample size in future studies will allow for a deeper evaluation and interpretation of dependence-associated alterations in the neurological compartment.

### 3.3. Detection thresholds and sensitivity analysis

The use of a stringent detection threshold (≥2/4 animals in one group, ≤1/5 in the comparison group) was employed to minimize false discoveries while maintaining sensitivity for biologically relevant changes. We subsequently performed a sensitivity analysis to confirm the impact on the findings and determined that these thresholds were capable of gleaning the most biological insight while accounting for technical variation and inter-individual variation of drinking and associated behaviors of the animals used in the study, which may have influenced protein detection (see Supplementary file 4, Tables S1 – S3). This approach identified 140 Dep-preferred and 62 Non-dep-preferred proteins, providing high-confidence biomarker candidates for validation studies. Post-hoc power analysis revealed that our study design (*n* = 4–5 per group) provided >99 % power to detect large effect sizes (detection rates of 100 % vs 0 %) but only 38 % power for moderate effects (75 % vs 20 % detection rates). This suggests that our findings likely underestimate the true number of proteins differing between groups, particularly those with moderate effect sizes. The limited *n* value in the current study underlines the necessity of performing a follow up validation study with a larger sample size, calculated to be *n* = 12–15/group for 0.8 power for the detection of moderate effect sizes and would also enable discernment of sex-specific differences that may provide further biological and mechanistic insight into the transition to alcohol dependence. This would also enable quantitative comparison between shared proteins, thus providing a more comprehensive analysis and understanding of alterations to the CSF proteome in AUD models. As protein classification is based on binary detection rather than continuous abundance measures, observed differences in detection frequency may partly reflect proteins near the detection threshold. This approach provides high-confidence identification of group-specific proteins but may not capture all abundance-based differences between groups.

### 3.4. IL-6R administration as a confounder

Given the increasing interest in elucidating the role of IL-6 in AUD (Vore and Deak, 2021) and in alcohol abstinence-related mechanical allodynia (Borgonetti et al., 2025; Borgonetti et al., 2023), in the current proteomics study we analyzed CSF obtained from IL-6R antibody treated animals; this also allowed us to determine whether alternative neuroinflammatory mechanisms are at play in non-dependent alcohol-exposure vs alcohol dependence. Importantly, the effects seen in the Dep group were not ameliorated by IL-6R treatment, suggesting that there are multiple pathways contributing to the dependence-associated neuroinflammation reported here. Dep mice presented with CSF signatures of BBB disruption, astrogliosis, and ER stress that were absent in non-dependent alcohol-exposed animals, despite both groups receiving IL-6R antibody. Incorporating 2 BCE drinking data, we found that BBB markers (MMP2, LAMB2) correlated with alcohol dose while astrogliosis (GFAP) correlated with dependence severity (escalation), suggesting mechanistically distinct pathologies (Supplementary file 4, Fig. S3). The persistence of these changes despite IL-6R blockade indicates IL-6-independent mechanisms underlie alcohol-induced CNS injury, with CX3CL1/fractalkine signaling as a candidate alternative pathway to be investigated in future studies. Although, as the levels of IL-6 were not directly measured during this study, we cannot rule out the role of IL-6 signaling in the neuroinflammatory cascades affecting BBB dysfunction prior to the IL-6R antibody administration.

To further investigate whether the rat anti-mouse IL-6R antibody (isoform 2B; IGG2B) used in our study was detectable in the mouse CSF samples, we searched the proteomics data against the *Rattus norvegicus* proteome and filtered the output to include peptides from the IGG2B protein (see Supplementary file 2 for the list of identified sequences filtered at 1 % FDR). Out of three identified peptide sequences belonging to IGG2B, one sequence was unique to rat (DILLISQNAK), which was identified in all CSF samples (Supplementary file 4, Fig. S1). Others have shown that intravenously administered antibodies can cross the BBB even when it is not compromised (Wang et al., 2018), and IgG antibody has been detected in the interstitial brain fluid in naïve mice by other methods (Le Prieult et al., 2021; Altendorfer-Kroath et al., 2023). This presents MS-based proteomics as a potential screening tool for detecting antibodies in the CNS following their peripheral applications in small volumes of CSF.

Of further note is the detection of classic IL-6 targets, CRP and SAA4, that are associated with acute inflammatory response in the Non-dep group. The absence of these proteins from Dep animals suggests a shift from acute phase response in Non-dep alcohol exposure to chronic neuroinflammation and CNS response in alcohol dependence. Other factors such as IL-1, TNF-α, and IL-1β can also upregulate CRP and SAA4, which further supports the hypothesis that there are alternative mechanisms responsible for the differential inflammatory profiles observed in Dep and Non-Dep groups.

### 3.5. Sex as a variable

While the current study lacked the statistical power to perform sex stratification, expanding this work to identify sex-specific differences in dependence-mediated molecular mechanisms of BBB dysfunction and immune dysregulation is warranted. Further considerations or expansions in this area of research may also include changes in abundance of histone deacetylases and are known to be affected in addiction models concerned with sexual dimorphism (Harris et al., 2016). This suggests that the transient and longer-term molecular mechanisms of addiction may be potentiated by epigenetic dynamics and is an ongoing area of research in the field of addiction (Renthal and Nestler, 2009; Jordi et al., 2013; Walker et al., 2015; Hamilton and Nestler, 2019). Future studies may focus specifically on histone post-translational modification (PTM) analysis and alterations to normal PTM patterns in males and females during the transition to alcohol dependence (Mews et al., 2019).

## 4. Conclusions

This study identified differences in the CSF proteomes between alcohol dep and non-dependent moderate alcohol drinking mice indicative of a shift from maintained protective mechanisms to neuroinflammation and loss of immune regulation in alcohol dependence. Proteins detected in the Non-dependent group support active protective processes, including complement regulation, anti-inflammatory signaling, and neuronal calcium homeostasis, which are lost or diminished during the transition to dependence. In contrast, the alcohol dependent-specific proteins indicate multiple pathological responses involving BBB breakdown, neuroinflammation, and cellular stress. This is one of the few studies to provide experimental evidence of BBB breakdown in a CIE-2 BCE model, although it has been acknowledged by others that acute and chronic alcohol exposure likely plays a role in compromised BBB function due to EtOH-induced alterations to endothelial tight junctions, cytokine release, transport receptors, blood flow, and angiogenesis (Vore and Deak, 2021). The contrast between the two proteomes in our study suggests that alcohol dependence involves a combination of pathological processes and loss of protective mechanisms. Importantly, our results agree with the proposed mechanisms of BBB breakdown mentioned above, whereby the detection of proteins involved in the neurovascular system such as endothelial cells (VE-cadherin), pericytes (ACTA), and basement membrane (LAMB2, fibulins) suggests that alcohol dependence affects all components of the BBB architecture. While our proteomic findings strongly suggest vascular compromise and BBB disruption based on the detection of endothelial junction proteins, basement membrane components, and pericyte markers in CSF, we acknowledge that CSF proteomics alone cannot definitively confirm functional barrier permeability changes. Future studies incorporating direct BBB permeability assays would provide complementary functional validation of these molecular findings.

Importantly, we provide a link to human studies that have detected two of the strong candidates identified here, MMP2 and BIP, in human serum and liver samples (Prystupa et al., 2015; Gaither et al., 2025), indicating that these putative markers of alcohol dependence detected in CSF may not be confined to the central nervous system. This presents an opportunity to explore alternative diagnostic routes of alcohol-dependence-related CNS damage via peripheral blood (Kornhuber et al., 1987) or tissue sampling in future studies.

Notably these proteomic data are in agreement with our recent studies (using the same CIE-2 BCE mouse model) showing that alcohol dependence causes a strong neuroimmune response in astrocytes in the nucleus accumbens, including activation of interferon and interleukin pathways, while moderate drinking alone caused homeostatic responses in astrocytes but did not show evidence of a neuroimmune activation (Hashimoto et al., 2025). While these findings provide preliminary evidence for distinct proteomic signatures in alcohol dependence, the identified proteins represent minimum estimates due to limited statistical power for moderate effect sizes. The identification of clinically validated biomarkers (GFAP, UCHL1, CHI3L1) in the dependence group suggests potential for developing diagnostic and monitoring tools for alcohol-related brain injury. Furthermore, the comprehensive nature of the pathological response, combined with the loss of protective mechanisms, indicates that alcohol dependence creates a vulnerable brain state that may predispose to accelerated aging and neurodegenerative processes. Validation in larger cohorts is essential to capture the full scope of dependence-associated molecular changes.

## 5. Methods

### 5.1. Animals

The male and female C57BL/6 J mice (*n* = 4 and 5; 29.2 ± 1.63 g and 22.4 ± 0.55 g, respectively) obtained from The Jackson Laboratory (Bar Harbor, ME) were housed in a temperature- and humidity-controlled room (12 h reverse light cycle) and provided with food and water ad libitum. All the animal procedures comply with the ARRIVE guidelines and were approved by The Scripps Research Institute (TSRI) Institutional Animal Care and Use Committee (IACUC #09–0006), consistent with the National Institutes of Health Guide for the Care and Use of Laboratory Animals.

### 5.2. Chronic intermittent ethanol – two bottle choice model

The mice used in this study were part of a cohort consisting of a total of 12 males and 12 females undergoing the chronic intermittent ethanol vapor-2 bottle choice paradigm (CIE-2 BCE) as described (Lopez and Becker, 2014; Patel et al., 2021; Patel et al., 2019; Warden et al., 2020) and treatment with the InVivoMAb anti-mouse IL-6R antibody (#BE0047, BioXCell).

**Fig. 6.**
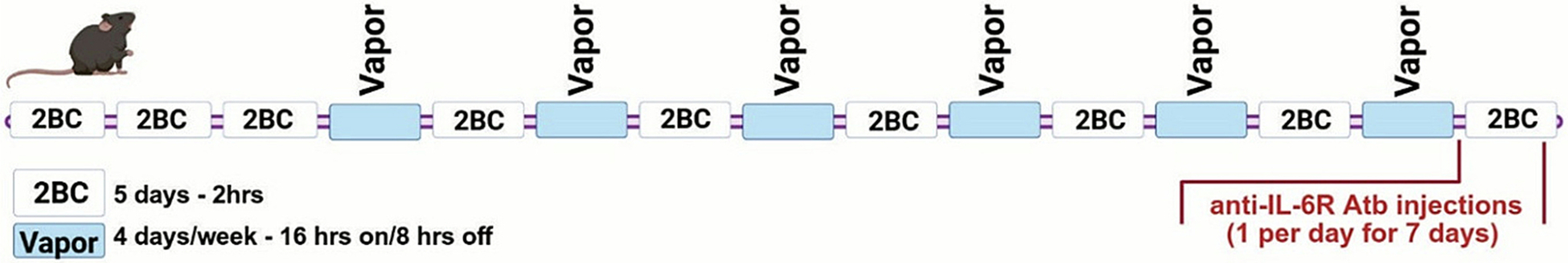
Schematic of the CIE-2 BCE paradigm.

Young adult male (*n* = 4) and female (*n* = 5) C57BL/6 mice were used in this study. Mice were 12 weeks old at the time of study commencement. To acclimate the mice to ethanol, they were singly housed and allowed 24 h access to 15 % ethanol in addition to their normal water and food for 4 days (Mon-Fri). After this, mice were returned to their original group housing. For an additional 15 days (5 days per week for 3 weeks) mice were singly housed for two hours with access to two drinking tubes, one containing 15 % ethanol and the other containing water (i.e. two bottle choice or 2 BCE) for 30 min before the lights were turned off. After this baseline drinking, the mice were assigned to the ethanol Non-dep or ethanol-dependent groups, with groups matched for baseline ethanol and water consumption. The ethanol Non-dep mice had a moderate level of voluntary ethanol consumption through limited access 2 BCE drinking sessions. In contrast, the ethanol-dependent mice received intermittent vapor exposure in addition to 2 BCE drinking sessions, resulting in the escalated voluntary ethanol intake. The mice underwent 6 cycles of 4 days of CIE (16 h ethanol vapor/8 h air) with their home cages inserted into vapor chambers (La Jolla Alcohol Research, La Jolla, CA) and 3 days forced abstinence followed by 5 days of 2 BCE (same parameters as in baseline training) and 2 days forced abstinence (Fig. 6). The Non-dep mice were exposed only to air in their home cages. Immediately before each ethanol vapor/air exposure, the dependent mice were injected (i.p.) with 1.75 g/kg ethanol +68.1 mg/kg pyrazole (Sigma, St Louis, MO) and Non-dep mice with 68.1 mg/kg pyrazole, as pyrazole is a competitive inhibitor of alcohol dehydrogenase and results in higher, more stable blood alcohol levels (Goldberg and Rydberg, 1969). The blood ethanol levels (BELs) were determined weekly using an Agilent 7820 A GC coupled to a 7697 A (headspace-flame-ionization) and were compared with and calibrated using a 6-point serial diluted calibration curve of 300 mg/dL ethanol (Cerilliant E-033). Results were used to adjust ethanol drip rates in the vapor chambers to achieve BELs of 150–250 mg/dL. (See Table 3 for blood alcohol levels measured in Dep mice during CIE-2BCE.)

The mice used in this study were injected with the InVivoMAb anti-mouse IL-6R antibody (BE0047, BioXCell) intraperitoneally (200 microgram/day) for 7 consecutive days. The first injection of the antibodies was 24 h after removal from the last vapor exposure. Thus, the antibody injections span over the forced WD after the vapor session and 4 days of a 2 BCE testing period. All injections were conducted to match the injection time during the 2 BCE testing period, 30 min before the drinking session. On the final day of antibody exposure CSF was collected between 2 and 4 h post injection.

### 5.3. CSF collection

Mice were 27.4 weeks of age at the time of CSF collection. After heavily anesthetizing the mice with isoflurane, the mice were secured in a stereotaxic unit, which continued to administer isoflurane. The area above the cisterna magna (located between the cerebellum and the dorsal surface of the medulla oblongata and receiving CSF from the 4th ventricle) was then isolated by cutting and deflecting the layers of skin and muscle at the back of the head. A pulled pipette was used to collect the cerebrospinal fluid by capillary pressure. Approximately 5 – 20 μl of cerebrospinal fluid was collected over no more than 30 min. and the mice were immediately euthanized. The collected CSF samples were stored at −80 °C until analyzed.

### 5.4. Proteomics sample preparation

Mouse CSF samples were processed for proteomic analysis using a modified filter-aided sample preparation protocol, as previously described (Turner et al., 2022). Briefly, samples (~5–15 μL) were solubilized in 20 μL of lysis buffer containing 1 % *w*/*v* sodium deoxycholate, 1 % w/v sodium dodecyl sulphate, 100 mM dithiothreitol (DTT) and 100 mM Tris-HCl pH 8.5, supplemented with protease inhibitors (Pierce Protease Inhibitor mini tablets, Thermo Scientific). Samples in lysis buffer were vortexed, heated to 95 °C for 3 min, and sonicated in a water bath for 5 min. Samples were incubated on ice for 10 min before being loaded onto Nanosep 30 K centrifugal filter devices (Cytiva) and centrifuged for 15 min at 14,000 x*g* and 21 °C until all the sample had passed through the filter. Samples were reduced with 25 mM DTT in 8 M urea for 1 h at RT, then centrifuged as in the previous step. Filters were washed once with 8 M urea, then 50 mM iodoacetamide in 8 M urea was added to the filters and incubated in the dark at RT for 20 min. Filters were centrifuged once and washed again with 8 M urea, followed by another wash with 100 mM ammonium bicarbonate pH 8–8.5 (AMBIC). Trypsin suspended in 100 mM AMBIC was added to each filter and an additional 60 μL of 100 mM AMBIC was added to each filter. The filter lids were left open, and samples incubated in a humidified chamber overnight at 37 °C. The next day, filters were transferred to clean 1.5 mL protein lobind tubes (Eppendorf) and centrifuged to elute peptides. One additional elution was performed by adding another 40 μL 100 mM AMBIC to the filters and repeating the centrifugation. Samples were acidified with formic acid (FA) to a final concentration of 0.1 %. A peptide assay was performed to determine peptide concentration following the manufacturer’s instructions (Pierce Colorimetric Quantitative Peptide Assay, Thermo Scientific). A volume equivalent to 350 ng peptides was loaded onto Evotips (Evosep) for peptide desalting and concentration, following the manufacturer’s instructions.

### 5.5. Liquid-chromatography tandem mass spectrometry (LC-MS/MS)

Data were acquired on a Fusion Lumos Tribrid Mass Spectrometer (Thermo Scientific) equipped with electrospray ionization source, coupled to a Evosep nano-LC system. Samples were run with a 15 spd (~88 min) method with a flowrate of 220 nL/min in DIA mode. Buffer A consisted of H_2_O/0.1 % FA and Buffer B was Acetonitrile/0.1 % FA (LC-MS grade, Fisher Scientific). Peptides were separated by reversed-phase HPLC on an in-house packed analytical capillary column, with dimensions 25 cm, 150 nm internal diameter, with BEH 1.7 μm C18 resin (Waters). Peptides were eluted from the column tip and nanosprayed into the mass spectrometer, with spray voltage set to 2 kV at the back of the column. Collision energy (CE) was fixed at 30 %, and peptides were fragmented with High-Energy Collisional Dissociation (HCD) in the orbitrap. Data were acquired with a 15 spd LC method (~88-min gradient) with a flowrate of 220 nL/min in DIA mode.

The DIA method consisted of 60 fixed-width, sequential isolation windows of width 10 *m*/*z* ranging from 300 to 900 *m*/*z* and performed in the quadrupole. Default charge state was +1, MS data type was set to profile and AGC target was set to 4e^5^ ions. Mass offset was set to 1.0005 with a 1 *m*/*z* overlap between windows. MS and MS/MS scans were performed in the orbitrap, with a resolution of 120 K and 15 K, respectively. MS/MS scans were performed over a range of 200–2000 *m*/*z*. Maximum injection time was 100 ms for MS and MS/MS scans. The MS/MS AGC target was set to 1e^6^ ions and data type was centroid.

Samples were run in a carefully randomized order to minimize systematic bias from group or sex. Prior to batch commencement, a HeLa commercial digest standard (Pierce^™^ HeLa Protein Digest Standard, cat no 88328, ThermoFisher Scientific) was run to verify optimal instrument performance. All samples were processed in a single batch to eliminate inter-batch variation. Given the small sample size and limited amount of material available, we relied on the inherent reproducibility of DIA acquisition rather than technical replicates, as DIA provides unbiased sampling of precursor ions and minimizes missing values.

## 6. Data analysis

Raw MS data were converted to ‘.dia’ format and searched in DIA-NN v 2.1.0 (Demichev et al., 2020) at 1 % FDR against an in silico predicted library generated using the *Mus musculus* reference proteome (downloaded from Uniprot on 3 March 2025; 21,803 sequences). The settings were as follows: Protease - Trypsin/P; Missed Cleavages – 1; Max number of mods – 3; N-term excision, C carbamidomethylation, Ox(M) were enabled; MBR was disabled; – -export-quant was enabled; all other settings were as default. The report file was loaded into R and the data were filtered to remove common contaminants (Cambridge Centre for Proteomics common Repository of Adventitious Proteins; CCP-cRAP) and further filtering at Q.Value, PG.Q.Value, and Global.Q.Value ≤0.01. The diann_maxlfq function from the R package ‘diann’ was used to obtain aggregated protein quantities from peptide information. For all comparisons, proteins were considered detected if quantified in ≥2 replicates within each group, unless otherwise stated. The R package ‘enrichR’ (v 3.4) was used for enrichment analysis (Kuleshov et al., 2016), and the online web tool for STRING protein network analysis (https://string-db.org/) was used to perform protein-protein interaction network analysis and generate all associated figures/plots. Post-hoc power analysis revealed >0.99 power for large effect sizes but only 0.38 power for moderate effects, indicating our protein lists represent minimum estimates of true group differences.

Shapiro-Wilk normality tests were performed to determine appropriate statistical testing for protein and peptide data. Peptide precursors and proteins identified in mouse CSF at 1 % FDR were subsequently compared using Student’s *t*-test for equal variance.

For identification of the rat IGG2B protein corresponding to the IL-6R antibody, we performed a separate DIA-NN search against the rat proteome (*Rattus norvegicus*; downloaded from Uniprot on 12 September 2024: 21,800 entries) with the same search settings. IGG2B peptide sequences were extracted from the rat search results, and three unique peptide sequences were identified (Supplementary file 2). The DILLISQNAK peptide was identified as unique to rat (i.e., non-homologous between mouse and rat), while the other two peptides were homologous between species. To visualize this peptide, it was copied into Skyline (Pino et al., 2020) and an in silico spectral library was generated within Skyline (v 25.1.0.237, MacCoss Lab, Department of Genome Sciences, University of Washington, WA) using koina (intensity model: Prosit_2020_intensity_HCD; iRT model: AlphaPept_rt_generic). All settings were set as default for centroid/orbitrap data, and the DIA window information was imported using one of the results files. All the results files were then imported into Skyline using a retention time (RT) tolerance of 10 min, and the spectra compared to the DIA-NN output for *m*/*z* and RT matching. The peak areas and associated information were extracted as confirmation of peptide identity (see Supplementary files 3 and 4).

## Supplementary Material

1

2

3

4

## Figures and Tables

**Table 1:** Dep-preferred proteins.

Protein	Function	Dep Detection	Non-dep Detection	Evidence Strength
MMP2	ECM degradation, BBB breakdown	4/4 (100 %)	0/5 (0 %)	Strong
CTL2A	Expressed in activated T-cells	4/4 (100 %)	0/5 (0 %)	Strong
PGS2	Involved in collagen fibril formation; belongs to damage-associated molecular pattern (DAMP) family	4/4 (100 %)	0/5 (0 %)	Strong
BIP	Molecular chaperone, protein quality control, BBB disruption	4/4 (100 %)	1/5 (20 %)	Strong
GFAP	Astrocyte activation marker	3/4 (75 %)	0/5 (0 %)	Strong
LAG3	Immune checkpoint protein	3/4 (75 %)	0/5 (0 %)	Strong
NPTX1	Synaptic plasticity regulator	3/4 (75 %)	0/5 (0 %)	Strong
CHI3L1	Astrocyte-derived inflammatory marker	3/4 (75 %)	1/5 (20 %)	Strong
ACTA	Pericyte contractile protein	3/4 (75 %)	1/5 (20 %)	Strong
LAMB2	Basement membrane component	3/4 (75 %)	1/5 (20 %)	Strong
CSPG5	Neural development	4/4 (100 %)	1/5 (20 %)	Strong
NFASC	Axon-glia interactions	4/4 (100 %)	1/5 (20 %)	Strong
RELN	Synaptic plasticity, neuronal migration	4/4 (100 %)	1/5 (20 %)	Strong
C1QA	Complement activation	2/4 (50 %)	0/5 (0 %)	Moderate
VCAM1	Endothelial adhesion molecule	2/4 (50 %)	0/5 (0 %)	Moderate
CADH5	VE-cadherin, endothelial junctions	2/4 (50 %)	1/5 (20 %)	Moderate
CX3CL1	Microglial chemokine	2/4 (50 %)	0/5 (0 %)	Moderate
UCHL1	Neuronal injury marker	2/4 (50 %)	1/5 (20 %)	Moderate

ACTA – α smooth Muscle Actin (Actin, aortic smooth muscle); BIP – Binding Immunoglobulin Protein (also known as GRP78/HSPA5); C1QA - Complement C1q A Chain; CADH5 - Cadherin 5 (VE-Cadherin); CHI3L1 - Chitinase 3 Like 1 (YKL-40); CTL2A - Protein CTLA-2-alpha; CSPG5 - Chondroitin Sulfate Proteoglycan 5 (Neuroglycan C); CX3CL1 - C-X3-C Motif Chemokine Ligand 1 (Fractalkine); GFAP - Glial Fibrillary Acidic Protein; LAG3 - Lymphocyte Activation Gene 3; LAMB2 - Laminin Subunit Beta 2; MMP2 - Matrix Metalloproteinase-2; NFASC - Neurofascin; NPTX1 - Neuronal Pentraxin 1; PGS2 - Decorin; RELN - Reelin; UCHL1 - Ubiquitin C-Terminal Hydrolase L1; VCAM1 - Vascular Cell Adhesion Molecule 1.

**Table 2:** Non-dep-preferred proteins.*

Protein	Function	Non-dep Detection	Dep Detection	Evidence Strength
TAGL3	Cytoskeletal regulation	4/5 (80 %)	0/4 (0 %)	Strong
KV5AB	Kappa light chain variable region	4/5 (80 %)	1/4 (25 %)	Strong
CALB1	Calcium-binding neuroprotection	4/5 (80 %)	1/4 (25 %)	Strong
LV1C	Lambda light chain variable region	3/5 (60 %)	0/4 (0 %)	Moderate
SUMO2/3	Protein modification, stress response	3/5 (60 %)	1/4 (25 %)	Moderate

CALB1 – Calbindin 1; KV5AB - Ig kappa chain V-V region HP R16.7; LV1C - Ig lambda-1 chain V region S178; SUMO2/3 - Small Ubiquitin-like Modifier 2/3; TAGL3 - Transgelin-3.

* Note: Proteins listed represent minimum estimates of group differences due to limited power for detecting moderate effect sizes.

**Table 3:** Summary of Blood Alcohol Levels (BAL) during CIE-2 BCE for EtOH-dependent mice.

	CIE 1	CIE 2	CIE 3	CIE 4	CIE 5	CIE 6	Overall
Male Mean	91.8	171.7	144.2	132.4	165.7	235.1	156.8
Female Mean	145.5	152.8	134.2	177.8	158.5	199.3	161.4
Combined Mean	118.7	162.3	139.2	155.1	162.1	217.2	**159.1**

Values are expressed as mg/dL.

## Data Availability

All data has been supplied as Supplementary files, or submitted to online repositories, as described in the ‘Data Availability’ section.

## Appendix A. Supplementary data

All R scripts used for data analysis and figure generation can be found at the GitHub repository https://github.com/NataliePTurner/MouseCSF. All MS data raw files and sample annotations list have been uploaded to the MassIVE repository (https://massive.ucsd.edu/ProteoSAFe/static/massive.jsp), with the dataset identifier C5GX4573B. The final filtered quantitative MS data file containing protein IDs, and quantities can be found as Supplementary File 1. Supplementary file 2 contains the list of IGG2B peptide sequences identified using the rat proteome and filtered at 1 % FDR. Supplementary file 3 contains the exported results from Skyline for the DILLISQNAK peptide. Supplementary file 4 contains histograms of the DILLISQNAK peak areas normalized to total area for each sample (S1), mouse drinking data from baseline through to CIE6 (S2), correlation plots of Dep-specific markers and CIE drinking and drinking escalation (S3), and a sensitivity analysis for condition-specific proteins (Dep and Non-dep; Table S1–S3). Supplementary data to this article can be found online at [https://doi.org/10.1016/j.nbd.2025.107254].
