# Supplementary material for: The effects of alcohol dependence on the CSF proteome in mice: Evidence for blood-brain barrier dysfunction and neuroinflammation
Source: Neurobiol Dis. Author manuscript; Available in PMC 2026 Feb 15. (PMC12906712; doi:10.1016/j.nbd.2025.107254)
Supplement: 4 [file NIHMS2145577-supplement-4.docx]

**
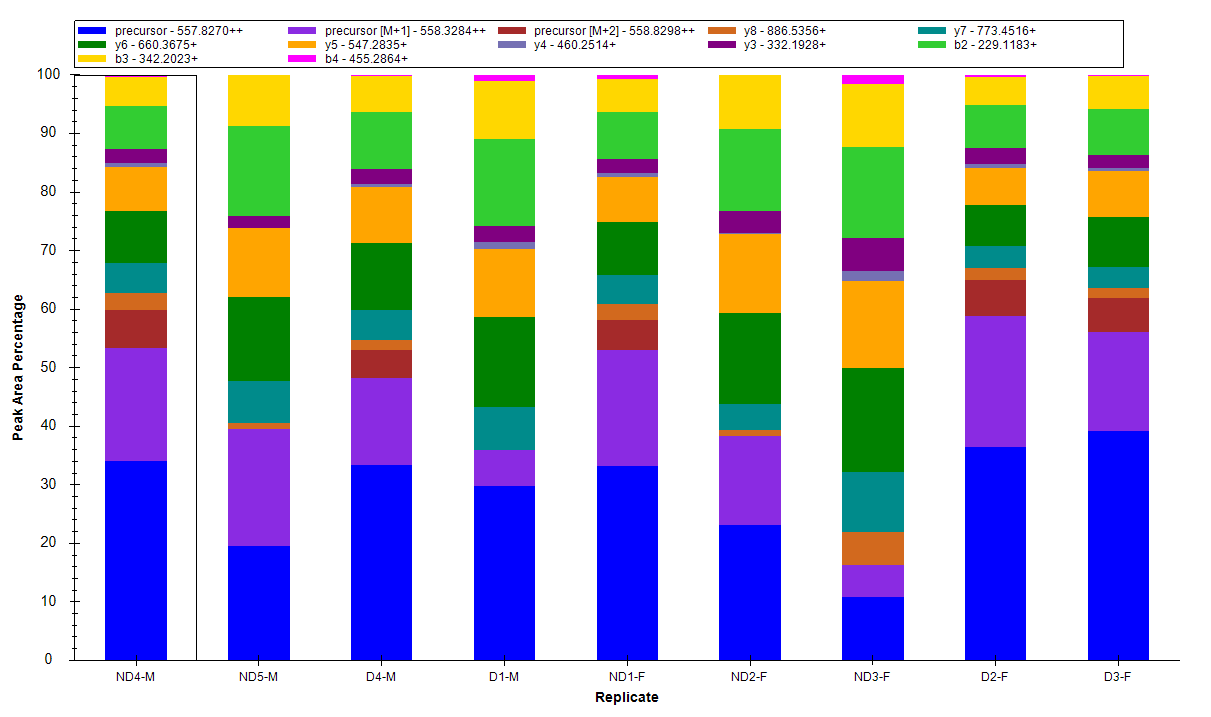
Figure S1:** Peak area percentages of the IGG2B_RAT peptide DILLISQNAK (normalized to total peak area). Legend indicates ion types.

**
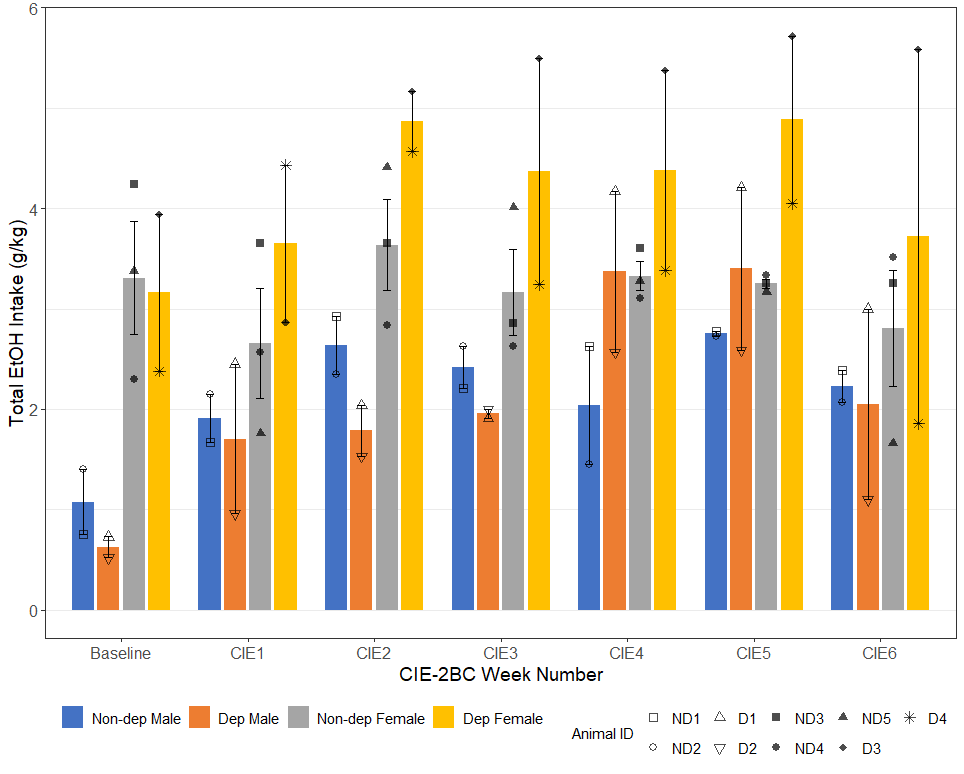
Figure S2:** Mouse drinking data from CIE-2BC model normalized to g alcohol (EtOH)/kg body weight. Bars represent mean, error bars ± SEM.

**
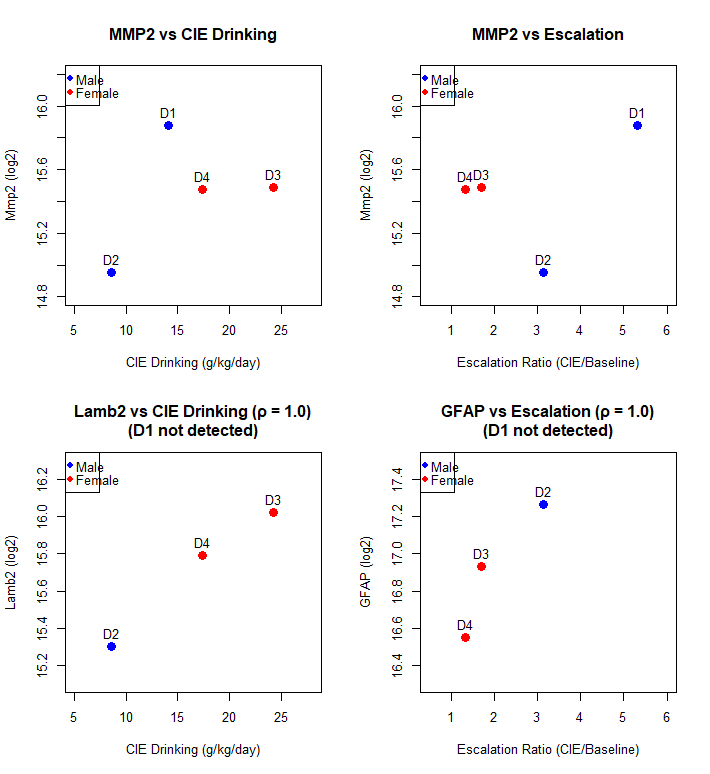
**

**Figure S3:** Trends between drinking behavior and log2 intensities of BBB/neuroinflammatory markers GFAP, LAMB2, and MMP2 in dependent animals (D).

**Table S1. Effect of Threshold Stringency on Protein Classification**

| **Threshold** | **Criteria** | **Dep-preferred** | **Non-dep-preferred** |
| --- | --- | --- | --- |
| **Strictest** | 100% preferred, 0% other | 3 | 0 |
| **Strict** | ≥75/80% preferred, 0% other | 6 | 1 |
| **Current Strong*** | ≥75/80% preferred, ≤20-25% other | 13 | 3 |
| **Current All*** | ≥50/60% preferred, ≤20-25% other | 18 | 5 |
| **Relaxed** | ≥50/60% preferred, ≤40-50% other | 18 | 5 |

** Thresholds used in this study. Dep-preferred: Strong ≥75% (3/4) Dep and ≤20% (1/5) Non-dep; Moderate ≥50% (2/4) Dep and ≤20% (1/5) Non-dep. Non-dep-preferred: Strong ≥80% (4/5) Non-dep and ≤25% (1/4) Dep; Moderate ≥60% (3/5) Non-dep and ≤25% (1/4) Dep.*

**Table S2. Robustness Categorization of Group-Preferential Proteins**

| **Robustness Tier** | **Detection Pattern** | **Proteins** |
| --- | --- | --- |
| **Tier 1**  **(Most Robust)** | 4/4 Dep, 0/5 Non-dep | MMP2, CTL2A, PGS2 |
| **Tier 2**  **(High)** | 4/4 Dep, 1/5 Non-dep | BIP, CSPG5, NFASC, RELN |
| **Tier 2**  **(High)** | 3/4 Dep, 0/5 Non-dep | GFAP, LAG3, NPTX1 |
| **Tier 3**  **(Moderate)** | 3/4 Dep, 1/5 Non-dep | CHI3L1, ACTA, LAMB2 |
| **Tier 3**  **(Moderate)** | 2/4 Dep, 0/5 Non-dep | C1QA, VCAM1, CX3CL1 |
| **Tier 4**  **(Borderline)** | 2/4 Dep, 1/5 Non-dep | CADH5, UCHL1 |
| **Tier 1**  **(Most Robust)** | 4/5 Non-dep, 0/4 Dep | TAGL3 |
| **Tier 2 (High)** | 4/5 Non-dep, 1/4 Dep | KV5AB, CALB1 |
| **Tier 3**  **(Moderate)** | 3/5 Non-dep, 0/4 Dep | LV1C |
| **Tier 4**  **(Borderline)** | 3/5 Non-dep, 1/4 Dep | SUMO2/3 |

*Proteins are categorized by robustness based on detection patterns. Tier 1 proteins survive the strictest thresholds (100% in preferred group, 0% in other). Tier 2 proteins meet high thresholds. Tier 3 proteins meet moderate thresholds. Tier 4 proteins meet current thresholds but are sensitive to stricter criteria.*

**Table S3. Sensitivity Matrix: Dep-preferred Protein Classification Across Thresholds**

| **Protein** | **Dep** | **Non-dep** | **4/4, 0/5** | **≥3/4, 0/5** | **≥3/4, ≤1/5** | **≥2/4, ≤1/5** |
| --- | --- | --- | --- | --- | --- | --- |
| **MMP2** | 4/4 | 0/5 | ✓ | ✓ | ✓ | ✓ |
| **CTL2A** | 4/4 | 0/5 | ✓ | ✓ | ✓ | ✓ |
| **PGS2** | 4/4 | 0/5 | ✓ | ✓ | ✓ | ✓ |
| **BIP** | 4/4 | 1/5 | ✗ | ✗ | ✓ | ✓ |
| **GFAP** | 3/4 | 0/5 | ✗ | ✓ | ✓ | ✓ |
| **LAG3** | 3/4 | 0/5 | ✗ | ✓ | ✓ | ✓ |
| **NPTX1** | 3/4 | 0/5 | ✗ | ✓ | ✓ | ✓ |
| **CHI3L1** | 3/4 | 1/5 | ✗ | ✗ | ✓ | ✓ |
| **ACTA** | 3/4 | 1/5 | ✗ | ✗ | ✓ | ✓ |
| **LAMB2** | 3/4 | 1/5 | ✗ | ✗ | ✓ | ✓ |
| **CSPG5** | 4/4 | 1/5 | ✗ | ✗ | ✓ | ✓ |
| **NFASC** | 4/4 | 1/5 | ✗ | ✗ | ✓ | ✓ |
| **RELN** | 4/4 | 1/5 | ✗ | ✗ | ✓ | ✓ |
| **C1QA** | 2/4 | 0/5 | ✗ | ✗ | ✗ | ✓ |
| **VCAM1** | 2/4 | 0/5 | ✗ | ✗ | ✗ | ✓ |
| **CADH5** | 2/4 | 1/5 | ✗ | ✗ | ✗ | ✓ |
| **CX3CL1** | 2/4 | 0/5 | ✗ | ✗ | ✗ | ✓ |
| **UCHL1** | 2/4 | 1/5 | ✗ | ✗ | ✗ | ✓ |

*Green (✓) indicates protein meets threshold criteria; red (✗) indicates protein does not meet threshold. Columns represent increasingly relaxed thresholds from left to right. Header format: minimum Dep detection, maximum Non-dep detection.*

**Threshold Justification**

The selected thresholds balance sensitivity and specificity given the sample sizes (*n* = 4 Dep, *n* = 5 Non-dep). This also accounts for inter-individual variation in the 2BC drinking behavior and water intake of the animals.

- Requiring ≥75% (3/4) detection in Dep ensures majority sample representation without being overly restrictive
- Allowing ≤20% (1/5) detection in Non-dep accounts for technical variation and sporadic detection
- Asymmetric thresholds (20% vs 25% for cross-group detection) reflect the different sample sizes
- Strictest thresholds (4/4 Dep, 0/5 Non-dep) would retain only 3 proteins, potentially missing biologically relevant candidates.
